# Indoxyl sulfate (IS)-mediated immune dysfunction provokes endothelial damage in patients with end-stage renal disease (ESRD)

**DOI:** 10.1038/s41598-017-03130-z

**Published:** 2017-06-08

**Authors:** Hee Young Kim, Tae-Hyun Yoo, Yuri Hwang, Ga Hye Lee, Bonah Kim, Jiyeon Jang, Hee Tae Yu, Min Chang Kim, Joo-Youn Cho, Chan Joo Lee, Hyeon Chang Kim, Sungha Park, Won-Woo Lee

**Affiliations:** 1 Department of Microbiology and Immunology, Seoul National University College of Medicine, Seoul, South Korea; 2 Cancer Research Institute, Seoul National University College of Medicine, Seoul, South Korea; 3 Division of Nephrology, Department of Internal Medicine, Yonsei University College of Medicine, Seoul, South Korea; 4 BK21Plus Biomedical Science Project, Seoul National University College of Medicine, Seoul, South Korea; 5 Department of Biomedical Sciences, Seoul National University College of Medicine, Seoul, South Korea; 6 Division of Cardiology, Cardiovascular Hospital, Yonsei University College of Medicine, Seoul, South Korea; 7 Department of Clinical Pharmacology and Therapeutics, Seoul National University College of Medicine and Hospital, Seoul, South Korea; 8 Department of Health Promotion and Disease Prevention, Severance Hospital, Seoul, South Korea; 9 Cardiovascular and Metabolic Diseases Etiology Research Center and Department of Preventive Medicine, Yonsei University College of Medicine, Seoul, Korea; 10 Ischemic/Hypoxic Disease Institute and Institute of Infectious Diseases, Seoul National University College of Medicine; Seoul National University Hospital Biomedical Research Institute, Seoul, South Korea

## Abstract

Progressive renal failure causes uremia-related immune dysfunction, which features a chronic inflammatory milieu. Given the central role of end-stage renal disease (ESRD)-related immune dysfunction in the pathogenesis of cardiovascular diseases (CVDs), much attention has been focused on how uremic toxins affect cellular immunity and the mechanisms underlying pathogenesis of atherosclerosis in ESRD patients. Here, we investigated the characteristics of monocytes and CD4^+^ T cells in ESRD patients and the immune responses induced by indoxyl sulfate (IS), a key uremic toxin, in order to explore the pathogenic effects of these cells on vascular endothelial cells. In ESRD patients, monocytes respond to IS through the aryl hydrocarbon receptor (AhR) and consequently produce increased levels of TNF-α. Upon stimulation with TNF-α, human vascular endothelial cells produce copious amounts of CX3CL1, a chemokine ligand of CX3CR1 that is highly expressed on CD4^+^CD28^−^T cells, the predominantly expanded cell type in ESRD patients. A migration assay showed that CD4^+^CD28^−^ T cells were preferentially recruited by CX3CL1. Moreover, activated CD4^+^CD28^−^ T cells exhibited cytotoxic capability allowing for the induction of apoptosis in HUVECs. Our findings suggest that in ESRD, IS-mediated immune dysfunction may cause vascular endothelial cell damage and thus, this toxin plays a pivotal role in the pathogenesis of CVD.

## Introduction

Progressive loss of renal function is strongly associated with aberrant immune responses. Uremia accompanying renal failure causes immune dysfunction characterized by the paradoxical coexistence of immune activation and immune suppression^2, 3^. In patients with ESRD, the elevated risk of cardiovascular diseases is closely linked to uremia-related immune activation, such as hypercytokinemia and inflammation. On the other hand, the impaired immune responses in these patients lead to increased susceptibility to infections, poor adaptive immune responses to standard vaccination procedures, and even enhanced risk of malignancies^4–6^. Of importance, the two leading causes of death in patients with ESRD are cardiovascular disease (CVD) and infection, and both pathologic processes are closely associated with uremia-related immune dysfunction^4, 7^.

The retention of uremic toxins and cytokines in patients with chronic kidney diseases (CKD) plays a critical role in the generation of oxidative stress and the proinflammatory milieu, which likely affect the composition and function of the cellular immune system^8–10^. In fact, it has been reported that CD14^+^CD16^+^ monocytes and CD4^+^ T cells lacking expression of the co-stimulatory molecule CD28 (hereafter, CD4^+^CD28^−^ T cells) are markedly expanded in the patients with ESRD^11–16^. A significant increase in both cell subsets is also observed in patients with various chronic inflammatory disorders including autoimmunity, further implicating their pathogenic roles^17–21^. Given that CVD is broadly recognized as a chronic immune-mediated inflammatory disease, attention has recently been focused on the contribution of expanded CD14^+^CD16^+^ monocytes and CD4^+^CD28^−^ T cells to the pathogenesis of this disease in ESRD patients^22^.

Among over 100 uremic toxins identified^23^, the presence of indoxyl sulfate (IS) and *p*-cresyl sulfate (PCS), originating from microbial fermentation of proteins in the gut, are closely associated with adverse outcomes in patients with renal failure. The dietary amino acids tryptophan and tyrosine are bacterially metabolized into indoles and *p*-cresol in the colon, respectively. After absorption, these are further converted into IS and PCS in the liver^24^. Patients with CKD show markedly higher serum IS and PCS levels than do healthy individuals. Together these toxins are largely responsible for progression of CKD and the development of CKD-related complications such as CVD^25^. A number of studies have focused primarily on IS-mediated endothelial dysfunction in order to elucidate its pathogenic role in CVD^26, 27^. Although the concept of uremia-related immune dysfunction is now well appreciated, little is known about the impact of IS on the cellular immune system and chronic inflammatory responses that can accelerate development and progression of CVD in ESRD patients. Thus, we hypothesized that IS, a major uremic toxin, causes aberrant responses of the cellular immune system and that these immunological defects contribute to adverse effects on vascular endothelial cells.

Here we demonstrate that ESRD patients have significantly higher serum IS and PCS levels compared with those of healthy controls. Moreover, IS, but not PCS, induces secretion of TNF-α by human monocytes through the aryl hydrocarbon receptor (AhR), and this induction is dramatically repressed by treatment with AhR inhibitors or by knockdown using AhR-targeted siRNA. Upon stimulation with TNF-α, human endothelial cells predominantly produce CX3CL1, a specific chemokine ligand of CX3CR1, which is highly expressed on CD4^+^CD28^−^ T cells. Of importance, ESRD patients have a markedly higher frequency of circulating cytolytic CD4^+^CD28^−^ T cells, which are significantly expanded under chronic exposure to TNF-α when compared with age-matched healthy controls (HCs). A migration assay revealed that CD4^+^CD28^−^ T cells are preferentially recruited by CX3CL1. Moreover, CD4^+^CD28^−^ T cells stimulated through their T cell receptors (TCRs) induced apoptosis of human endothelial cells, suggesting that IS-mediated immune dysfunction could be associated with the development and accelerated progression of CVD in patients with renal failure.

## Results

### Indoxyl sulfate (IS), a uremic toxin, induces production of TNF-α by human monocytes

**Figure 1:**
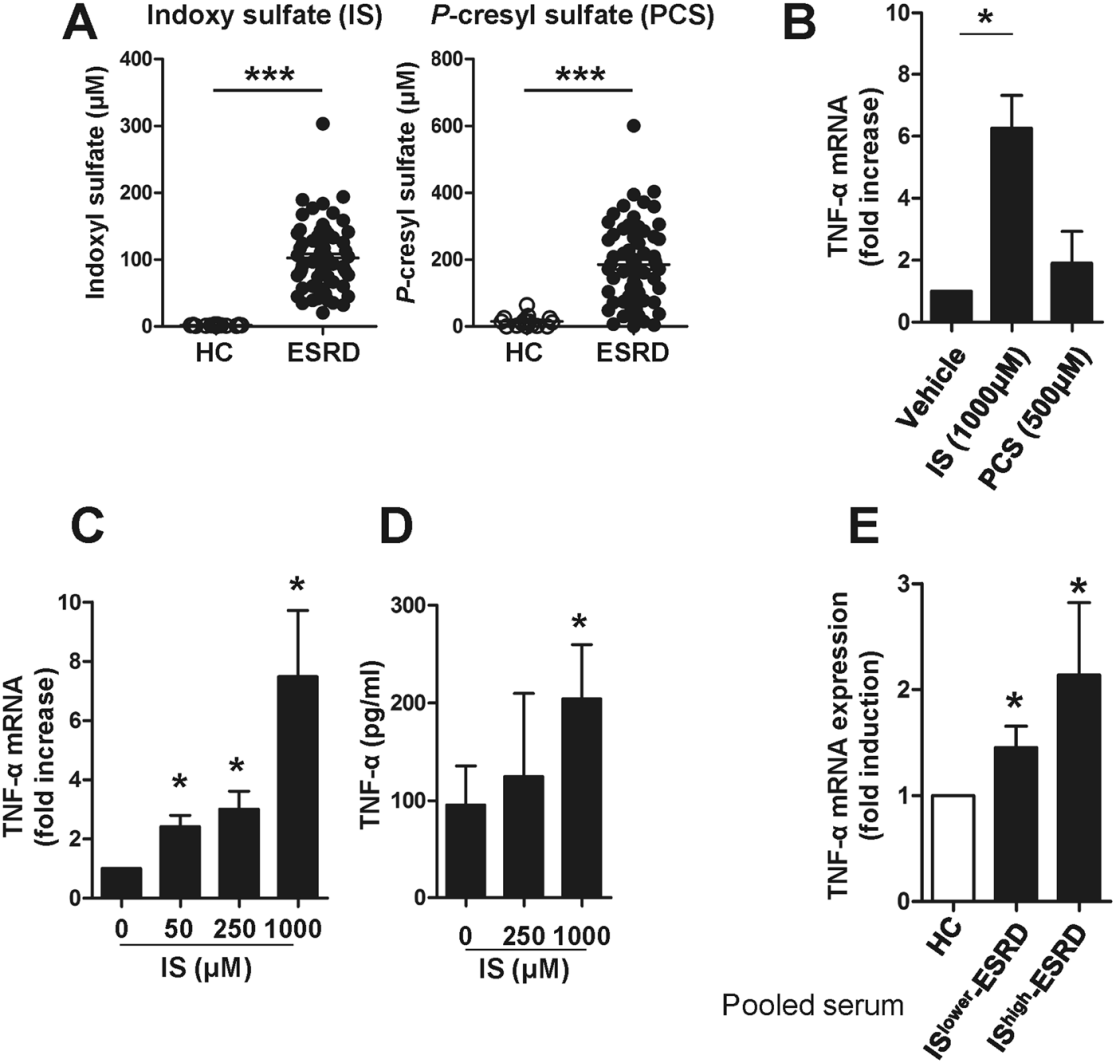
Indoxyl sulfate (IS), a uremic toxin, induces production of TNF-α by human monocytes. (**A**) Serum indoxyl sulfate (IS) and *p*-cresyl sulfate (PCS) levels in ESRD patients (n = 64) and healthy controls (n = 15) were quantified using liquid chromatography–tandem mass spectrometry (LC–MS/MS). (**B**) Purified monocytes were stimulated with 1,000 μM of IS or 500 μM of PCS for 24 hr and then TNF-α mRNA expression was analyzed by real-time RT-PCR. (**C** and **D**) Purified monocytes were stimulated with IS at the indicated concentrations. The expression of TNF-α mRNA was analyzed by real-time RT-PCR after a 24 hr stimulation (**C**) and its protein level was quantified by ELISA at 48 hr post-stimulation (**D**). (**E**) Sera were pooled from patients with the top 10 (IS^higher^-ESRD) and bottom 10 (IS^lower^-ESRD) IS serum concentrations, respectively. As a control, sera from healthy controls were pooled. Monocytes isolated from healthy controls were treated with 30% (v/v) of the indicated sera for 24 hr and TNF-α mRNA expression was analyzed by real-time RT-PCR. Expression of β-actin was used as a normalization control. Bar graphs and scatter plot show the mean ± SEM of three (**B**), five to seven (**C** and **D**), and four (**E**) independent experiments. *p < 0.05, **p < 0.01, and ***p < 0.001 by two-tailed unpaired (**A**) or paired *t*-test (**B**,**C**,**D** and **E**).

Retained uremic toxins are believed to be an underlying cause of the proinflammatory cytokine milieu seen in ESRD^1^. It has been demonstrated that protein-bound uremic toxins, indoxyl sulfate (IS) and *p*-cresyl sulfate (PCS), originating from microbial fermentation of proteins in the gut, are responsible for adverse outcomes in patients with renal failure^28, 29^. As expected, the concentrations of IS and PCS in plasma of ESRD patients (clinical characteristics of patients and HCs are presented in Table 1) were greatly higher than those in HCs (Fig. 1A, HC 1.87 ± 0.21 μM vs. ESRD 102.44 ± 6.26 μM for IS, *p* < 0.0001; HC 15.04 ± 4.7 μM vs. ESRD 185.41 ± 14.99 μM for PCS, *p* < 0.0001). The maximal levels of IS and PCS in ESRD patients were over 300 μM and 600 μM, respectively.

**Table 1:** Clinical characteristics of ESRD patients and healthy control subjects.

	ESRD (n = 50)	Control (n = 28)	*p*-value
Clinical variables			
Age (years)	53.2 ± 12.8	52.9 ± 9.1	0.910
Male gender	32 (64.0%)	18 (64.3%)	1.000
CAD	9 (18.0%)	0 (0%)	0.023*
Hypertension	38 (82.6%)	5 (17.9%)	<0.001*
DM	14 (31.1%)	1 (3.6%)	0.006*
Dyslipidemia	6 (13.0%)	6 (21.4%)	0.352
Current Smoker	5 (10.0%)	6 (21.4%)	0.188
BMI (kg/m^2^)	23.6 ± 3.4	24.0 ± 2.3	0.566
SBP (mmHg)	149.4 ± 22.6	119.3 ± 9.5	<0.001*
DBP (mmHg)	81.7 ± 11.4	77.3 ± 7.9	0.078
Anuria	35 (70%)		
Hemodialysis	22 (44%)		
Peritoneal dialysis	28 (56%)		
Dialysis duration (year)	5.5 ± 6.3		
Laboratory variables			
WBC count (×10^3^/μL)	6.3 ± 2.0	6.1 ± 1.5	0.566
Hemoglobin (g/dL)	11.0 ± 1.5	14.5 ± 1.8	<0.001*
Glucose (mg/dL)	102.0 ± 38.9	95.1 ± 17.0	0.380
Total cholesterol (mg/dL)	157.9 ± 33.4	193.5 ± 29.2	<0.001*
HDL cholesterol (mg/dL)	46.8 ± 17.3	53.0 ± 12.6	0.105
Triglyceride (mg/dL)	97.2 ± 51.3	149.3 ± 159.3	0.104
Uric acid (mg/dL)	6.1 ± 1.6	4.9 ± 1.3	0.002*
hsCRP (mg/L)	3.1 ± 6.8	1.0 ± 0.9	0.074
BUN (mg/dL)	58.3 ± 23.6	15.1 ± 4.1	<0.001*
Creatinine (mg/dL)	10.6 ± 3.7	1.0 ± 0.2	<0.001*
Albumin (g/dL)	3.9 ± 0.5	4.6 ± 0.2	<0.001*
Calcium (mg/dL)	9.1 ± 0.7		
Phosphorus (mg/dL)	5.0 ± 1.5		
Calcium-phosphorus product (mg^2^/dL^2^)	45.6 ± 13.9		

ESRD, end-stage renal disease; CAD, coronary artery disease; DM, diabetes mellitus; BMI, body mass index; BUN, blood urea nitrogen; SBP, systolic blood pressure; DBP, diastolic blood pressure; WBC, white blood cell; HDL, high-density lipoprotein; hsCRP, high-sensitivity C-reactive protein. Data are presented as mean ± SD or n (%). *A *p*-value < 0.05 was considered statistically significant.

Aberrant functional and phenotypical features of monocytes have been well reported in ESRD patients^1^. Consistent with previous studies^14, 15^, the frequencies of proinflammatory CD16^+^ monocytes as well as total monocytes (defined as HLA-DR^+^CD14^dim/+^ cells) in ESRD patients were significantly increased compared with those of HCs (Suppl. Fig. 1A and B). Given the central role of proinflammatory monocytes as drivers of vascular inflammation in CVD, we sought to investigate effect of IS and PCS on immune response of monocytes in ESRD.

We first investigated the induction of proinflammatory cytokines by IS and PCS in monocytes, and found augmented TNF-α mRNA (6.26-fold increase, Fig. 1B: *p* < 0.05) after a 24-hour treatment with IS, but not PCS. In agreement with the dose-dependent enhancement of its gene expression (Fig. 1C), the amount of TNF-α (204.01 ± 55.63 pg/ml) in the monocyte culture supernatant was significantly increased following 48 hours of IS treatment compared to the untreated group (95.55 ± 39.99 pg/ml) (Fig. 1D). It should be noted that apoptosis was not induced by treatment with the highest concentration (1,000 μM) of IS for 48 hr (data not shown). We also confirmed that IS induced increases in IL-1β mRNA in cells and IL-1β protein in supernatants after a 48-hour treatment with IS, but not PCS (Suppl. Fig. 2A and B). However, no induction of IL-6 mRNA and its protein was observed in monocytes with both IS and PCS treatment (Suppl. Fig. 3).

Consistent with previous reports^30, 31^, the serum of ESDR patient had significantly increased levels of TNF-α compared with those of HCs (Suppl. Fig. 4). To further investigate these findings, we selected patients with the top 10 (IS^higher^-ESRD) and bottom 10 (IS^lower^-ESRD) patients levels of serum IS, respectively, and pooled their sera into high and low groups for further analysis. Monocytes treated with 30% (v/v) of pooled sera for 24 hours upregulated TNF-α expression slightly, but significantly (IS^higher^-ESRD (n = 10; 184.08 ± 44.75 μM of IS) and IS^lower^-ESRD (n = 10; 38.10 ± 8.03 μM of IS)), compared with monocytes treated with pooled sera from HCs (n = 9) (Fig. 1E).

Taken together, these findings suggest that IS is involved in the induction of proinflammatory cytokines such as TNF-α and IL-1β productions by monocytes.

### IS-induced TNF-α expression is dependent on aryl hydrocarbon receptor (AhR)-mediated responses in human monocytes

**Figure 2:**
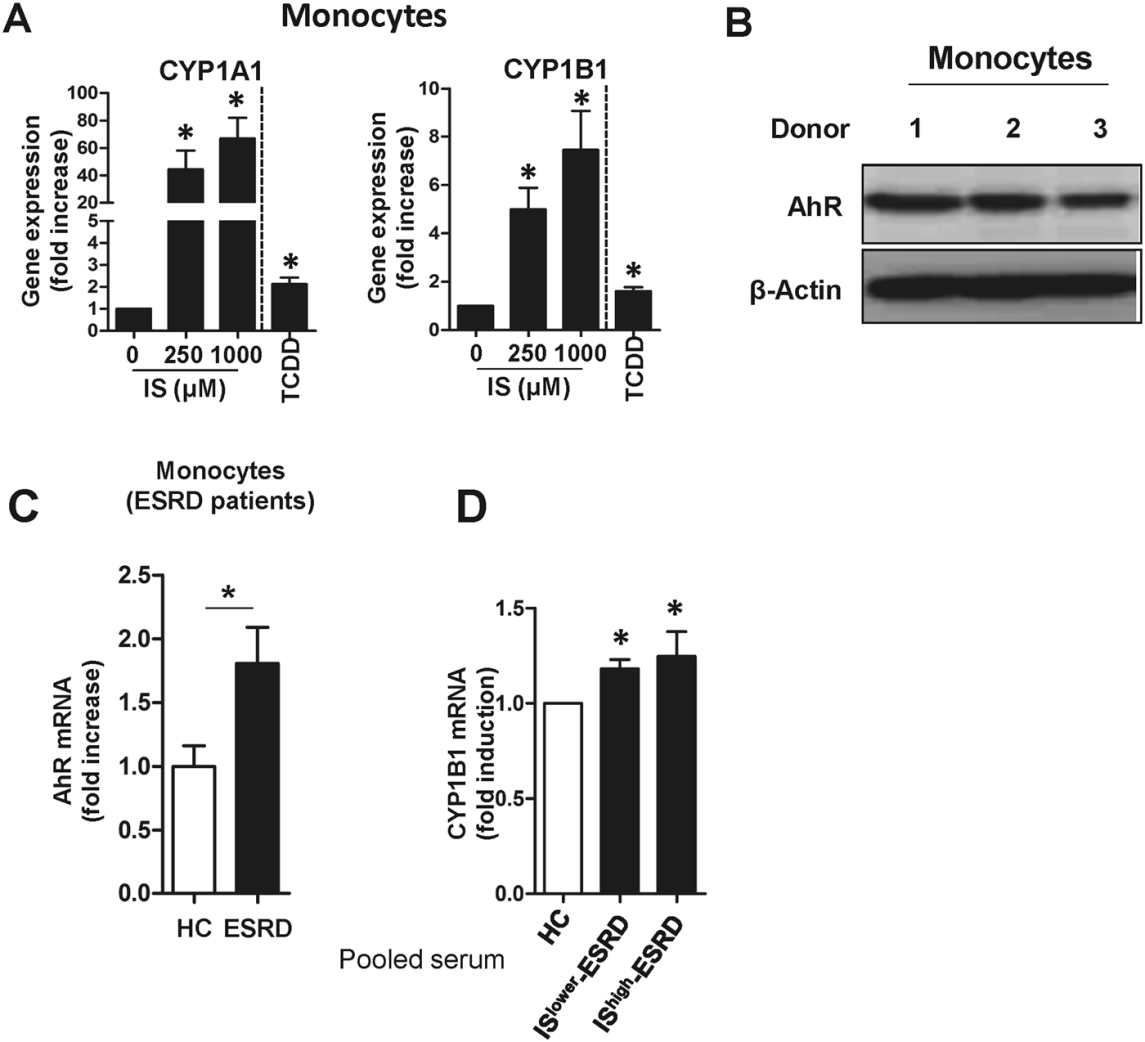
Indoxyl sulfate (IS) is a potent agonist for the AhR on human monocytes. **(A)** Purified monocytes from HCs were treated with IS at the indicated concentration for 24 hr, and the expression of AhR responsive genes CYP1A1 (left panel) and CYP1B1 (right panel) were analyzed by real-time RT-PCR. TCDD was used as a positive agonist control. **(B)** Western blot analysis of AhR in monocytes freshly isolated from peripheral blood of HCs (n = 3). **(C)** CD14^+^ monocytes were freshly isolated from ESRD patients (n = 4) and age-matched HCs (n = 4) and the expression of AhR mRNA was analyzed by real-time RT-PCR. **(D)** Monocytes isolated from healthy controls were treated with 30% (v/v) of the indicated sera for 24 hr and CYP1B1 mRNA expression was analyzed by real-time RT-PCR. Expression of β-actin was used as a normalization control (**A, C** and **D**). Bar graphs show the mean ± SEM of five (**A**) or four (**D**) independent experiments. *p < 0.05: compared to no treatment group and HCs by two-tailed paired (**A** and **D**) and unpaired *t*-test (**C**), respectively.

We next sought to explore how IS affects the inflammatory response of monocytes, which exhibit prominent ESRD-related changes. Since IS was recently identified as a potent endogenous ligand for the aryl hydrocarbon receptor (AhR)^32, 33^, responsiveness to IS was assessed by the expression of two genes known to be regulated by this pathway, CYP1A1 and CYP1B1. As shown in Fig. 2A, IS led to markedly enhanced expression of CYP1A1 and CYP1B1 mRNAs in monocytes (66.93 ± 15.08-fold increase; *p* < 0.012 and 7.45 ± 1.61- fold increase; *p* < 0.017, respectively). Of note, this induction was 5 to 30 times greater than that seen in cells treated with 2,3,7,8-Tetrachlorodibenzo-*p*-dioxin (TCDD), a potent AhR agonist. Western blot analysis confirmed that AhR was constitutively expressed at high levels by freshly-isolated monocytes (Fig. 2B), suggesting that circulating monocytes may be a major cellular subset responding to IS in the serum of ESRD patients. Furthermore, AhR mRNA expression was upregulated in monocytes of ESRD patients compared to that of HCs (Fig. 2C). In addition, treatment with pooled sera from ESRD patients (at 30% (v/v) for 24 hr) increased the mRNA expression of CYP1B1 in HC monocytes (Fig. 2D; *p* < 0.05).

**Figure 3:**
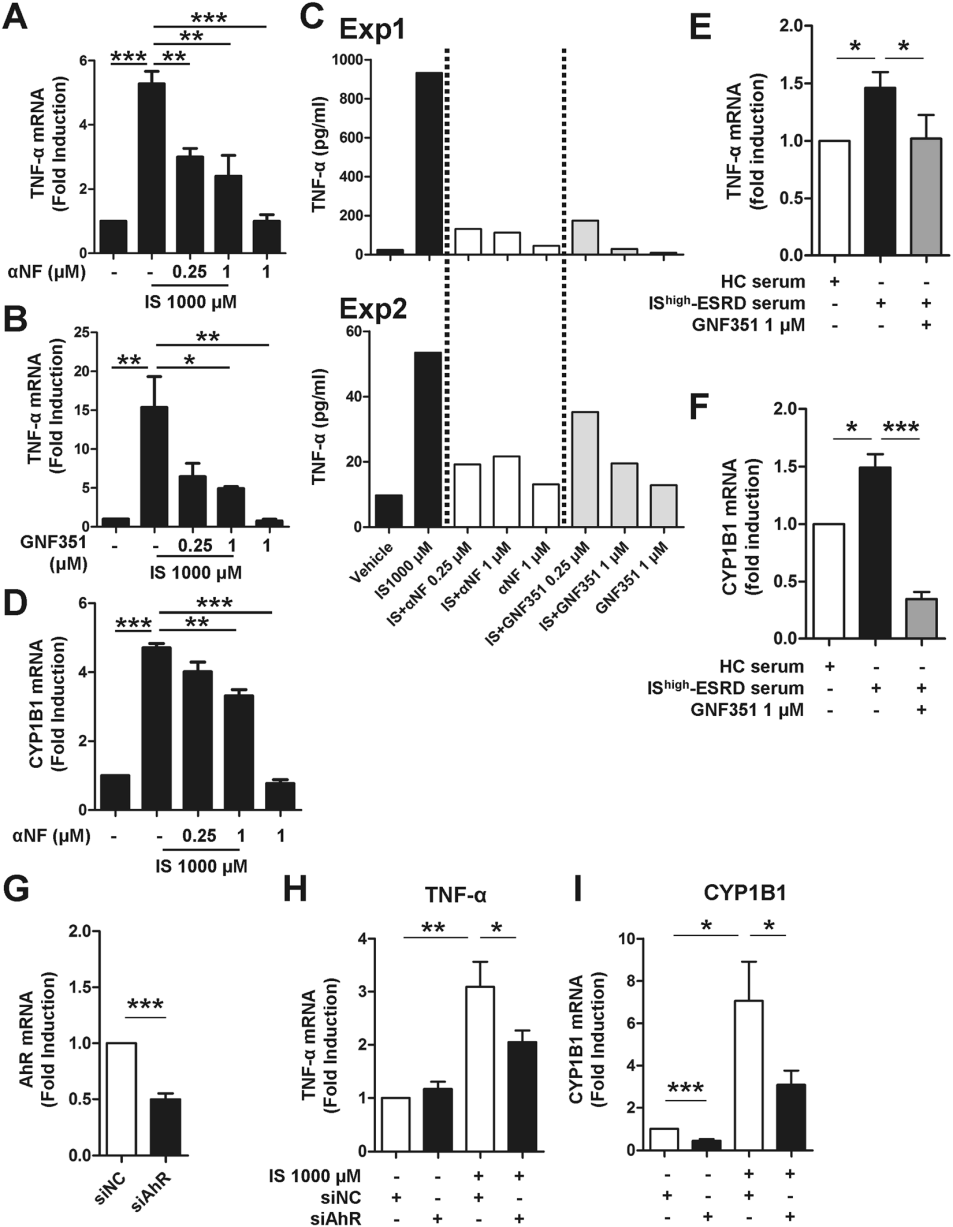
IS-induced TNF-α expression is regulated through AhR activation in human monocytes. Monocytes were co-treated with IS and AhR antagonists, α-NF or GNF351, at the indicated concentration for 24 or 48 hr. (**A** and **B**) At 24 hr post-treatment, TNF-α mRNA expression was analyzed by real-time RT-PCR. (**C**) At 48 hr post-treatment, the amount of TNF-α in the culture supernatant was quantified by ELISA. Two representative experiments of five are shown. (**D**) At 24 hr post-treatment, CYP1B1 mRNA expression was analyzed by real-time RT-PCR. Expression of β-actin was used as a normalization control. (**E** and **F**) Sera were pooled from patients with the top 10 (IS^higher^-ESRD) IS serum concentrations. As a control, sera from healthy controls were pooled. Monocytes isolated from healthy controls were treated with 30% (v/v) of the indicated sera for 24 hr with or without 1 μM of GNF351. Gene expression levels were analyzed by real-time RT-PCR. (**G**) Knockdown efficiency of AhR. Freshly purified total monocytes were transfected with AhR-specific or control siRNA (20 pmol of both siRNAs). AhR mRNA expression was analyzed at 24 hr after transfection by real-time RT-PCR. (**H** and **I**) siRNA transfected monocytes were treated with IS for 24 hr. Real-time RT-PCR was performed for analysis of TNF-α and CYP1B1 mRNA expression. Expression of β-actin was used as a normalization control. Bar graphs show the mean ± SEM of five (**A**) or four (**B** and **D**) or six (**E**–**I**) independent experiments. *p < 0.05, **p < 0.01, and ***p < 0.001 by one way ANOVA (**A**,**B** and **D**) and by two-tailed paired *t*-test (**E–I**), respectively.

To further investigate a role of AhR for IS-induced TNF-α expression, monocytes were stimulated with IS in the presence of potent AhR antagonists, α-naphthoflavone (α-NF) or GNF351. As shown in Fig. 3, IS-induced TNF-α mRNA and protein expression were significantly inhibited by both α-NF and GNF351 (Fig. 3A–C). As expected, α-NF inhibited expression of the AhR responsive gene, CYP1B1, (Fig. 3D), indicating that α-NF represses AhR-mediated responses in monocytes. In addition, blocking the AhR-mediated response with GNF351 completely abrogated the inductions of TNF-α, CYP1B1, and IL-1β by HC monocytes treated with pooled sera from ESRD patients (Fig. 3E and F and Suppl. Fig. 5).

Our finding was corroborated by an AhR siRNA (siAhR) knockdown experiment (Fig. 3G–I). Freshly purified human monocytes were transfected with AhR-targeted siRNA and then treated with IS for 24 hr. siAhR led to downregulation of its mRNA expression by approximately 50% compared with cells transfected with control siRNA (siNC) (Fig. 3G). Further, knockdown of AhR resulted in decreased TNF-α and CYP1B1 mRNA expression at around 50% in response to IS treatment compared to the control siRNA group (Fig. 3H and I).

Increased oxidative stress in patients with uremia is closely related to the pro-inflammatory state of the immune system^8, 34^. To examine whether IS induces ROS production in monocytes and its production affects TNF-α induction, we measured ROS production by IS-treated total monocytes. Monocyte significantly enhanced ROS production as early as 30 min after treatment with IS and its level increased until 2-hour of treatment (Suppl. Fig. 6A). IS-mediated ROS production was partially abrogated by ROS inhibitor NAC (N-acetyl-L-cysteine). Of important, AhR antagonist GNF351 had no effect on IS-mediated ROS production in primary monocytes (Suppl. Fig. 6B), suggesting that different mechanisms may be involved in increased level of ROS and enhanced production of TNF-α by IS-treated monocytes.

Taken together, these findings demonstrate that the AhR-mediated response is responsible for IS-induced TNF-α expression in human monocytes.

### Human endothelial cells substantially induce CX3CL1 in response to proinflammatory cytokine stimulation

**Figure 4:**
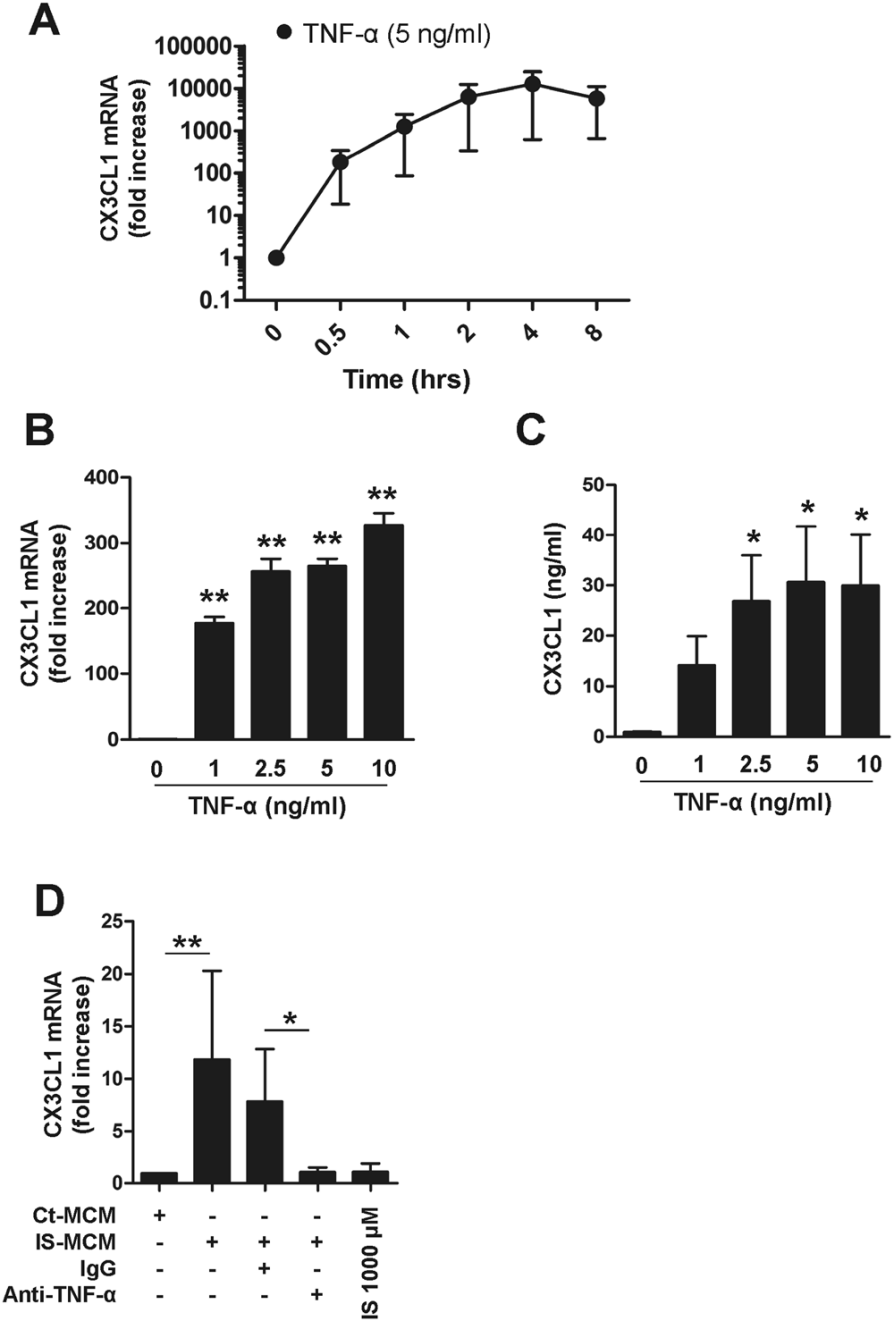
TNF-α markedly upregulates CX3CL1 production by HUVECs. (**A**) HUVECs were stimulated with TNF-α (5 ng/ml) up to 8 hours and CX3CL1 mRNA expression was analyzed by real-time RT-PCR at the indicated time-points. (**B**) HUVECs were treated with various concentrations of TNF-α (1 to 10 ng/ml) for 4 hours, and the expression of CX3CL1 was analyzed by real-time RT-PCR. (**C**) HUVECs were stimulated with various concentrations of TNF-α (1 to 10 ng/ml) for 18 hours and the amount of CX3CL1 in the culture supernatant was quantified by conventional ELISA. (**D**) Purified monocytes were treated with or without IS for 48 hr, and the supernatant (MCM: monocyte-conditioned media) of each culture was harvested. Control or IS-treated monocyte-conditioned media (Con- or IS-MCM) was added to confluent, cultured HUVECs in the presence of anti-TNF-α Ab or control IgG, followed by a 4 hr incubation. CX3CL1 mRNA expression in treated HUVECs was analyzed by real-time RT-PCR. Expression of β-actin was used as a normalization control. Bar graphs show the mean ± SEM of three to four independent experiments. *p < 0.05 and **p < 0.01: compared to no TNF-α treatment group by two-tailed paired *t*-test (**B** and **C**).

Selective recruitment of inflammatory immune cells is a critical step in the pathogenesis of cardiovascular diseases^35^. Because the chemokine CX3CL1 is known to play a cardinal role in plaque formation through recruitment of T cells and monocytes^36^, we investigated whether IS-induced proinflammatory cytokines lead to secretion of CX3CL1 by HUVECs. As seen in Fig. 4, CX3CL1 mRNA was immensely upregulated in HUVECs following stimulation with TNF-α. We also found that IL-1β induced marked upregulation of CX3CL1 mRNA in HUVECs (Suppl. Fig. 7). The upregulation of CX3CL1 mRNA in HUVECs was initiated as early as 30 minutes and reached a maximum level at 4 hours after TNF-α exposure (Fig. 4A). In addition, CX3CL1 gene expression increased in a dose-dependent manner in response to TNF-α with concentrations ranging from 0 to 10 ng/ml (327.00 ± 18.19-fold increase at 10 ng/ml TNF-α; *p* < 0.01), as did CX3CL1 protein production; however, CX3CL1 protein reached a plateau at 5 ng/ml of TNF-α (Fig. 4B and C). To further investigate this finding in a more physiological setting, purified monocytes were treated with IS for 48 hr and the culture supernatant [henceforth known as monocyte-conditioned media (MCM)] was added to HUVECs for 4 hr. Treatment of HUVECs with IS-treated MCM resulted in the marked upregulation of CX3CL1 mRNA, which was completely inhibited by TNF-α neutralizing antibody (Fig. 4D). In contrast, no induction of CX3CL1 mRNA was observed in HUVECs following treatment with IS alone. These findings reveal that the proinflammatory cytokine milieu, including TNF-α, produced by IS-stimulated monocytes is capable of inducing increased secretion of CX3CL1 by HUVECs. Moreover, the production of CX3CL1 may result in the selective recruitment of immune cells expressing CX3CR1, the chemokine receptor for CX3CL1.

### ESRD patients have higher numbers of CD4^+^CD28^−^ T cells preferentially expressing CX3CR1

**Figure 5:**
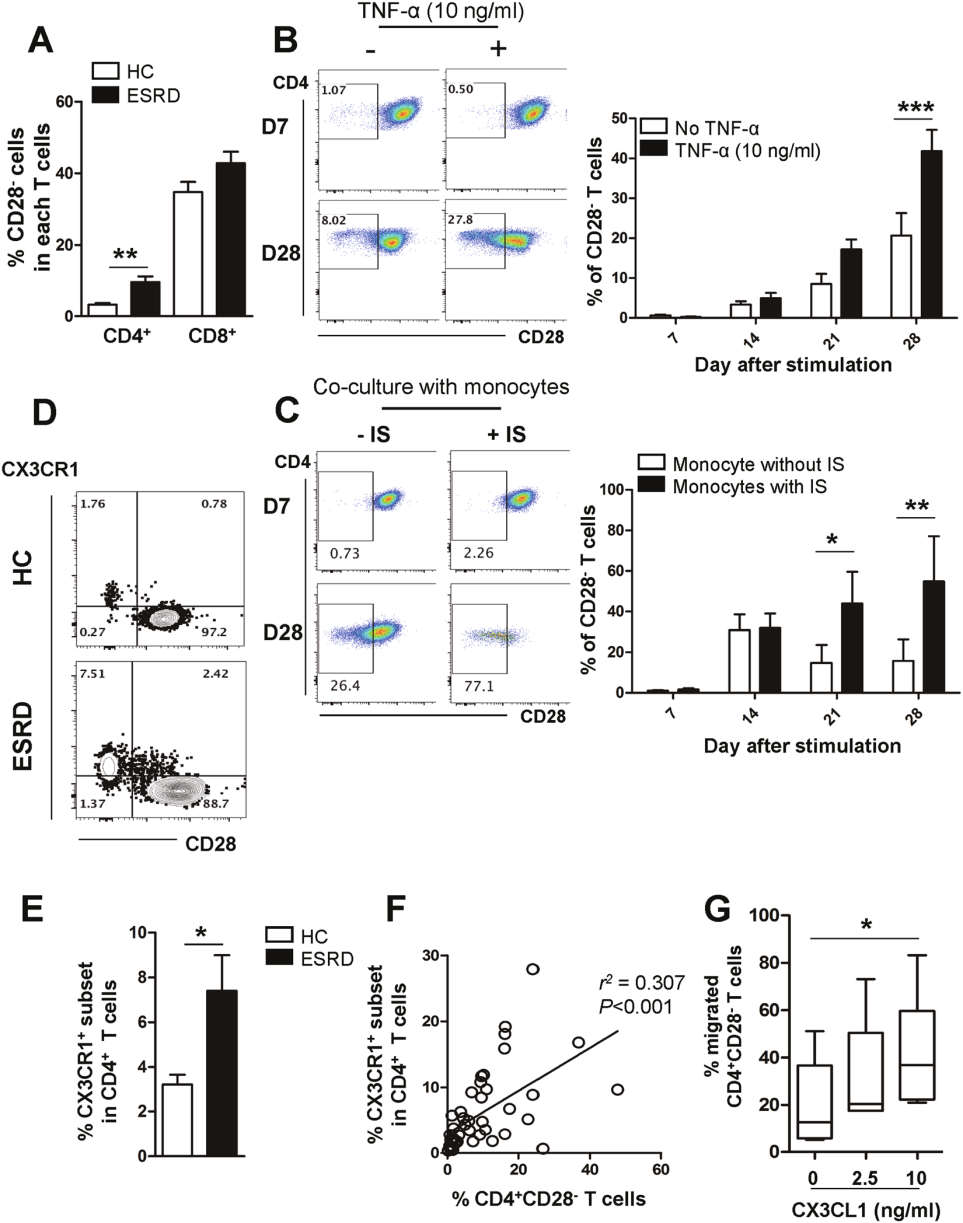
CD4^+^CD28^−^ T cells expressing CX3CR1, a receptor for CX3CL1, are expanded under the TNF-α rich environment in ESRD patients. (**A**) Frequencies (%) of CD28^−^ cells in CD4^+^ and CD8^+^ T cells in ESRD patients (n = 50) and age-matched HCs (n = 28). (**B**) Purified CD4^+^CD28^+^ cells from ESRD patients were stimulated with α-CD3/CD28 Ab-coated beads and IL-2 in the absence or presence of TNF-α. At 4 days, beads were removed using a magnet and the cytokines were re-supplemented every 3–4 days. CD28 expression was analyzed every 7 days by flow cytometry. Representative FACS plot of change in CD28 expression on cultured CD4^+^CD28^+^ T cells in ESRD patients under indicated culture conditions (Left). On the indicated day, cultured cells were harvested and the frequency of CD28^−^ cells was determined by flow cytometry (n = 4) (Right). (**C**) Purified naive CD4^+^ T cells were stimulated with α-CD3/CD28 Ab-coated beads and IL-2. At 4 days, beads were removed using a magnet and the cell were co-cultured with monocytes, which were stimulated with IS (1,000 μM) for 24 hr. IS-stimulated CD14^+^ monocytes were re-supplemented every 3–4 days. CD28 expression was analyzed every 7 days by flow cytometry. Representative FACS plot of change in CD28 expression on cultured naive CD4^+^ T cells under indicated culture conditions (Left). On the indicated day, cultured cells were harvested and the frequency of CD28^−^ cells was determined by flow cytometry (n = 3) (Right). (**D**) Representative contour plot of CX3CR1 expression on CD4^+^CD28^+^ and CD4^+^CD28^−^ T cells from ESRD patients and HCs (**E**) Expanded CX3CR1^+^CD4^+^ T cells in patients with ESRD compared with HCs. (**F**) Frequency (%) of CX3CR1^+^ cells positively correlates with the frequency of CD28^−^ cells in CD4^+^ T cells of ESRD patients (n = 46). Each data point represents an individual subject. (**G**) Freshly-purified CD4^+^ memory T cells from ESRD patients were stained with APC-conjugated anti-CD28 mAb and a chemotaxis assay was performed at various concentrations of CX3CL1 (0 to 10 ng/ml) for 2 hours using a transwell system. The frequency (%) of CD28^−^ T cells in migrated cells at various concentrations of CX3CL1 was analyzed by flow cytometry. Bar graphs show the mean ± SEM. *p < 0.05, **p < 0.01, and ***p < 0.005 by two-tailed unpaired *t*-test (**A** and **E**) or 2 way ANOVA test (**B** and **C**). *P* value in (**F**) was obtained using the Pearson correlation analysis. Box plots displaying medians, 25th and 75th percentiles as boxes, and minimum and maximum values as whiskers (n = 6). *p < 0.05 by two-tailed paired non-parametric test (**G**).

In agreement with the previous reports^11, 12^, ESRD patients exhibited markedly expanded CD28^−^ cells in the CD4^**+**^, but not in the CD8^**+**^, T cell compartment (Fig. 5A; *p* < 0.01 and *p* = 0.10, respectively). These expanded CD4^**+**^CD28^−^ cells in ESRD patients were attributable to significant accumulation of the CD45RA^**+**^ effector memory (EM) subset, which is comprised of terminally-differentiated cells. These cells arise through extensive cell divisions caused by repeated stimulation and are characterized by loss of co-stimulatory molecules, such as CD27, and gain of inhibitory, immune-regulatory molecules (Supplementary Fig. 8A and B)^37, 38^. In addition, CD4^**+**^CD28^−^ T cells exhibit a phenotype typical of dysfunctional senescent T cells that have experienced extensive cell divisions, including gains of CD57 and CD85j, and a loss of the IL-7 receptor α chain (Suppl. Fig. 8C). ESRD patients had significantly higher levels of serum TNF-α compared to healthy controls (Suppl. Fig. 4). Moreover, IS-treated monocytes produced an enhanced amount of TNF-α (Fig. 1D and C). Repeated antigenic stimulation under TNF-α exposure causes loss of CD28 on CD4 T cells under chronic inflammatory conditions^39^. Therefore, highly purified CD4^+^CD28^+^ T cells from ESRD patients were stimulated with anti-CD3/CD28 mAbs and cultured with recombinant human TNF-α for 28 days. In this long-term culture system, a significant increase in CD4^+^CD28^−^ T cells was found in the TNF-α treatment group after 28 days (Fig. 5B). To emphasize the link between IS-activated monocytes and the generation of CD4^+^CD28^−^ T cells, CD4^+^CD28^+^ naive T cells were stimulated with anti-CD3/CD28 mAbs for 3 days and co-cultured with IS-treated monocytes for another 25 days. A significant increase in CD4^+^CD28^−^ T cells was found after 21 days in the co-culture with IS-activated monocytes which produce a higher TNF-α (Fig. 5C). These data suggest that the TNF-α rich environment in ESRD patients leads to an accumulation of circulating CD4^+^CD28^−^ T cells.

The findings in Fig. 4 prompted us to examine whether ESRD patients have a higher frequency of immune cells expressing CX3CR1, the receptor for CX3CL1, and to determine which cell subsets express CX3CR1. Of note, CX3CR1 was predominantly expressed on CD4^+^CD28^−^ T cells, but not on CD4^+^CD28^+^ T cells, suggesting that the increased frequency of the CX3CR1^+^ subset in CD4 T cells is secondary to the significant accumulation of CD4^+^CD28^−^ T cells in ESRD patients (Fig. 5D). Flow cytometric analysis showed that the frequency of CD4^+^ T cells expressing CX3CR1 in ESRD patients was augmented more than two-fold over that of HCs (7.41 ± 1.59 vs. 3.21 ± 0.45; *p* < 0.04) (Fig. 5E). Moreover, the mutually exclusive expression pattern of CD28 and CX3CR1 in CD4 T cells was confirmed by the finding that the percentage of CD4^+^CD28^−^ T cells in ESRD patients correlates significantly with the percentage of CD4^+^CX3CR1^+^ T cells (Fig. 5F; *p* < 0.001).

To investigate the functional role of CX3CR1 expressed on CD4^+^CD28^−^ T cells, a transwell chemotaxis assay using recombinant CX3CL1 was performed with CD4 T cells derived from ESRD patients (Fig. 5G). The results clearly demonstrate that the frequency of migrating CD4^+^CD28^−^ T cells significantly increased up to the 10 ng/ml concentration of CX3CL1, indicating that T cells expressing CX3CR1 were preferentially recruited by recombinant CX3CL1.

Taken together, CD4^+^CD28^−^ T cells, which are greatly expanded under TNF-α-rich conditions in ESRD patients, preferentially express CX3CR1 conferring the unique ability to migrate toward CX3CL1 produced by activated vascular endothelial cells.

### CD4^+^CD28^−^ T cells have cytotoxic and senescent features that induce endothelial cell damage

**Figure 6:**
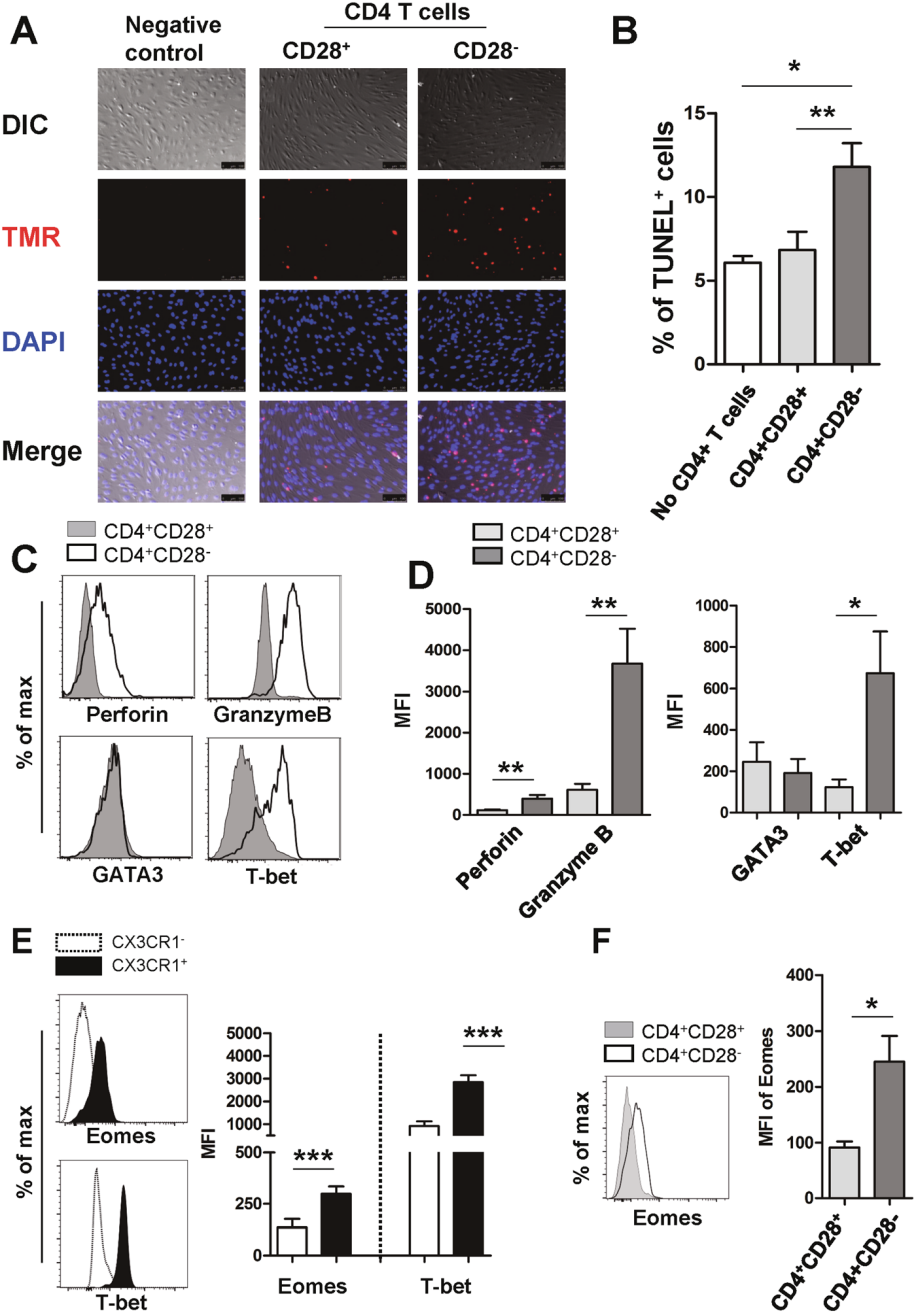
CD4^+^CD28^−^ T cells have features typical of cytotoxic T cells and induce death in HUVECs in response to TCR stimulation. (**A**) Activated CD4^+^CD28^−^ T cells induce death of HUVECs. HUVECs were pre-treated with IFN-γ (2,000 U/ml) for 48 hours and co-cultured with purified CD4^+^CD28^−^ T cells or CD4^+^CD28^+^ T cells in the presence of superantigen, SEB (10 ng/ml), and TSST-1 (10 ng/ml) for TCR stimulation. The level of cell death was analyzed by TUNEL assay. The nuclei in TUNEL^+^ apoptotic cells were detected by TMR (red) and DAPI (blue) was used for nuclei staining of HUVECs. Data is representative of four independent experiments. At least three images were analyzed in each group. Scale bar equals 100 μm. (**B**) Frequencies (%) of apoptotic cells among three treatment groups. (**C**) Representative histogram plot of cytotoxic granules (perforin and granzyme B) and transcription factors (GATA3 and T-bet) in CD4^+^CD28^+^ and CD4^+^CD28^−^ T cells from ESRD patients. (**D**) MFIs (mean fluorescent intensities) of cytotoxic granules and transcription factors were compared between CD4^+^CD28^+^ and CD4^+^CD28^−^ T cells (n = 11) (**E**) Representative histogram plot (Left panel) and MFIs (Right panel) of transcription factors, Eomes and T-bet in CX3CR1^+^ and CX3CR1^−^ CD4^+^ T cells from ESRD patients (n = 5). (**F**) Representative histogram plot (Left panel) and MFIs (Right panel) of Eomes in CD4^+^CD28^+^ and CD4^+^CD28^−^ T cells from ESRD patients (n = 5). *p < 0.05, **p < 0.01 and ***p < 0.005 by two-tailed paired *t*-test.

Our data show that TNF-α-stimulated human endothelial cells produce a considerable level of CX3CL1, which selectively recruits CD4^+^CD28^−^ T cells expressing CX3CR1. To investigate whether these CD4^+^CD28^−^ T cells have an adverse effect on vascular endothelial cells, HUVECs were stimulated with IFN-γ for 48 hours to induce MHC class II on their surface and were then co-cultured with ESRD patient-derived CD4^+^CD28^−^ T cells and CD4^+^CD28^+^ T cells in the presence of superantigen to crosslink TCR and MHC class II. Damage to co-cultured HUVECs was evaluated using the TUNEL apoptosis assay. As seen in Fig. 6A and B, the frequency of apoptotic HUVECs increased when co-cultured with CD4^+^CD28^−^ T cells compared with CD4^+^CD28^+^ T cells, suggesting that CD4^+^CD28^−^ T cells have the potential to invade and harm human endothelial tissue. Several human studies have suggested that cytotoxic CD4^+^ T cells are characterized by a loss of CD27 and CD28 surface expression and concomitant gain of CD57. Indeed, this phenomenon was also observed as shown in Supplementary Fig. 8C ^40^. In addition, in ESRD patients the expanded CD4^+^CD28^−^ T cells had significantly higher levels of cytotoxic granules, such as granzyme B and perforin, than CD4^+^CD28^+^ T cells had (Fig. 6C and D). Flow cytometric analysis revealed that CD4^+^CD28^−^ T cells predominantly expressed transcription factors typical of a Th1 profile (Fig. 6C and D), and thus, presumably would express large amounts of IFN-γ and TNF-α on activation. Recent studies reported that Eomesodermin (Eomes) plays a critical role in the development of long term memory and cytotoxic CD4 T cells^41, 42^. As expected by Figs 5F and 6D, both CX3CR1^+^ and CD4^+^CD28^−^ T cells in ESRD patients had a significantly higher Eomes expression compared with each counterpart subset (Fig. 6E and F). Our data suggest that the increased CD4^+^CD28^−^ T cells in ESRD patients display cytotoxic features suggesting they are capable of inducing endothelial cell damage.

## Discussion

**Figure 7:**
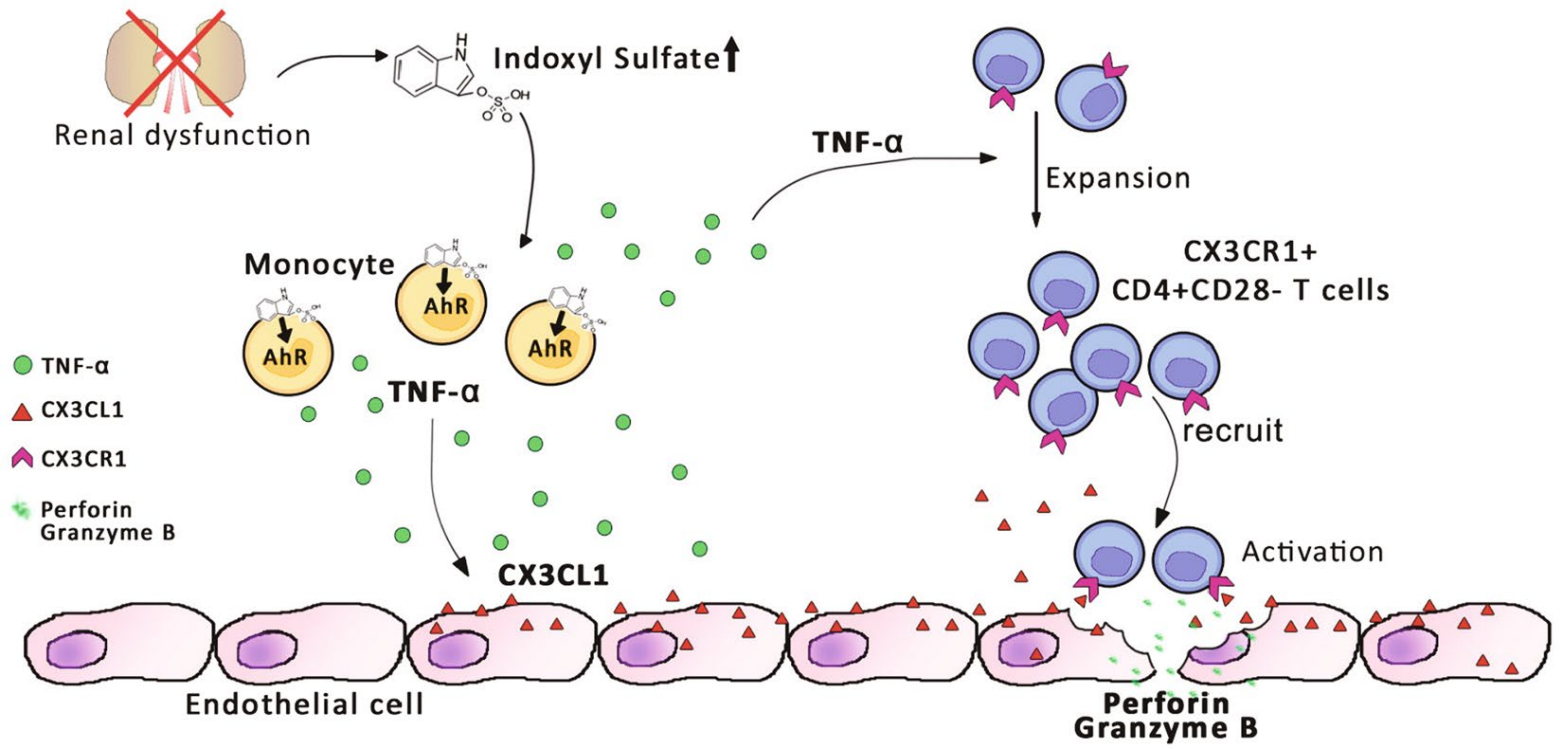
Proposed model of IS-mediated immune dysfunction provoking endothelial damage in ESRD patients. IS, a key uremic toxin which is dramatically accumulated in patients with chronic renal dysfunction, induces secretion of TNF-α by human monocytes through the aryl hydrocarbon receptor (AhR). Upon stimulation with TNF-α, human endothelial cells predominantly produce CX3CL1, a specific chemokine ligand of CX3CR1, which is highly expressed on CD4^+^CD28^−^ T cells. ESRD patients have a markedly higher frequency of circulating cytolytic CD4^+^CD28^−^ T cells, which are significantly expanded under chronic exposure to TNF-α. These CD4^+^CD28^−^CX3CR1^+^ T cells are preferentially recruited by CX3CL1 and induce apoptosis of human endothelial cells upon TCR activation.

In the present study, we demonstrate ESRD-related alterations of monocytes and CD4 T cells in patients and their putative pathogenic roles for endothelial damage, which is a critical step for the development and accelerated progression of CVD in patients with ESRD. *In vitro* culture data clearly demonstrate that IS, a key uremic toxin, functions as an endogenous stimulus that cause monocytes to produce augmented amounts of TNF-α through the AhR-mediated pathway. Further, stimulation with TNF-α leads to production of copious amounts of CX3CL1 by human vascular endothelial cells and gradual loss of CD28 molecules by CD4 T cells under TCR stimulation. Of note, CD4^+^CD28^−^ T cells, the predominantly accumulated cell type in peripheral blood of ESRD patients, preferentially express CX3CR1, a chemokine receptor for CX3CL1. These CD4^+^CD28^−^ T cells possess cytotoxic capability when activated including the ability to induce apoptosis of human endothelial cells, as well as the ability to preferentially migrate in response to CX3CL1 (Fig. 7).

ESRD is associated with significant increases in cardiovascular disease, which accounts for considerable morbidity and mortality and may be induced by the uremic proinflammatory milieu^43^. Uremia accompanying chronic renal failure has a profound impact on the immune system including increased numbers, composition, and functions of various immune cells^2, 3, 44^. In ESRD patients, uremia mediates a vicious cycle between oxidative stress and the inflammatory immune response exacerbating a chronic proinflammatory milieu, which exerts detrimental effects on both the innate and adaptive immune systems and consequently increases the risk of atherosclerotic disease^8^. However, the mechanisms underlying uremic toxin-mediated immune dysfunction and its pathogenic roles are still unclear.

In agreement with a previous report^1^, our data clearly show expansion of CD16^+^ monocytes in ESRD patients (Suppl. Fig. 1). CD16^+^ monocytes play a pivotal role in the pathophysiology of many inflammatory diseases^45^. Considering that CD14^+^CD16^+^ monocytes potently induce TNF-α, IL-1β, and IL-6 in response to various stimuli^46^, expanded CD14^+^CD16^+^ monocytes in ESRD patients might contribute to the generation of the proinflammatory milieu. Moreover, CD14^dim^CD16^+^ monocytes have the unique ability to patrol the blood vessel endothelium for signs of damage and infection^46^.

As seen in Fig. 1A, serum levels of the endogenous uremic toxins IS and PCS are dramatically elevated due to inadequate renal clearance in patients with CKD (102.44 ± 6.26 μM and 185.41 ± 14.99 μM, respectively) compared with healthy individuals (1.87 ± 0.21 μM and 15.04 ± 4.70 μM, respectively)^47^. Furthermore, these toxins cannot be efficiently removed by hemodialysis due to their strong binding capacity for serum proteins^48^. Many *in vitro* experiments have demonstrated that IS and PCS harm endothelial cells and vascular smooth muscle cells in chronic kidney disease and also inhibit proliferation and wound repair of vascular endothelial cells^49^. However, due to their constant exposure, immune cells may also be major targets of these toxins. In fact, in a mouse model it has been found that IS stimulates macrophage function and enhances inflammatory responses associated with LPS^50^. Furthermore, diminished serum concentrations of IS due to treatment with AST-120, an orally-administered intestinal sorbent, lessens monocyte inflammation and attenuates the progression of atherosclerosis in a CKD animal model^34^. In the present study, we demonstrate that monocytes preferentially respond to IS and secrete increased amounts of the proinflammatory cytokine TNF-α (Fig. 1D). Clinically, endogenous IS diluted in 30% patient serum is sufficient to induce TNF-α mRNA in monocytes derived from healthy controls. Therefore, IS may be responsible for elevated serum TNF level in patients with ESRD (Suppl. Fig. 4 and Fig. 1E). IS was recently identified as a potent endogenous ligand for the aryl hydrocarbon receptor (AhR)^32^. AhR was initially known as the dioxin receptor and is a ligand-activating transcription factor involved in biological detoxification responses against a variety of ligands including environmental pollutants^51–54^. However, recent studies demonstrated that there are many naturally occurring AhR ligands such as tryptophan derivatives (e.g. indoxyl sulfate)^51, 53^. Given the potential involvement of tryptophan-derived uremic toxins and AhR-activating pollutants in cardiovascular diseases, defining the AhR-mediated pathway is important for understanding pathogenesis of cardiovascular diseases^51, 55^.

Like dendritic cells and macrophages^56^, circulating human monocytes constitutively express a high level of AhR (Fig. 2B). Further, interruption of the AhR-IS interaction using AhR antagonists and AhR siRNA led to a significant diminution of TNF-α mRNA and its protein level in human monocytes/macrophages (Fig. 3), clearly supporting an important role of AhR-mediated responses in pathogenesis of ESRD patients. A recent study reported that TCDD, a potent AhR ligand, induced TNF-α production in a human macrophage cell line via the AhR-EGFR-ERK pathway^57^. However, a negative regulatory role for AhR against LPS-induced inflammatory responses of macrophages (but not those induced by other TLR ligands) has also been suggested, as increased IL-6 and TNF responsiveness has been demonstrated in AhR knockout, murine macrophages^58^. Therefore, more detailed studies are needed in order to clarify how IS-elicited AhR activation influences production of proinflammatory cytokines by circulating monocytes.

Besides overproduction of cytokines, recent studies have shown that uremic monocytes also induced angiotensin converting enzyme (ACE) and ROS production, which plays a crucial role in inflammation and progression of atherosclerosis^8, 34, 59, 60^. IS-activated monocytes significantly increased ROS production (Suppl. Fig. 6) and showed a tendency to increase the expressions of ACE, angiotensin II receptor type 1 and 2 (AT1R and AT2R) (data not shown). However, AhR antagonist GNF351 had no effect on IS-mediated ROS production as well as ACE, AT1R, and AT2R in primary monocytes (Suppl. Fig. 6 and data not shown), suggesting that different mechanisms are involved in these changes and enhanced production of TNF-α by IS-treated monocytes.

The gene signature of vascular endothelial cells is largely changed in the context of the inflammatory cytokine milieu^61^. In particular, TNF-α treatment has been shown to predominantly induce the expression of certain chemokine genes, such as CXCL2, CXCL6 and CX3CL1, by HUVECs. These chemokines play crucial roles in leukocyte recruitment toward sites of inflammation^62^. Consistent with previously-reported microarray data^62^, our data show that TNF-α markedly induces production of CX3CL1 by HUVECs (Fig. 4). Of note, CX3CR1, the chemokine receptor for CX3CL1, is predominantly expressed by CD4^+^CD28^−^ T cells, which are greatly expanded in ESRD patients (Fig. 5A and D). In addition, we show CD4^+^CD28^−^ T cells possess a unique capability to migrate toward activated vascular endothelial cells producing CX3CL1 (Fig. 5G). The accumulation of CD28^−^ T cells, mainly CD8 T cells and to a lesser degree CD4 T cells, was initially regarded as an age-related change (so called immunosenescence)^37^; however, recent studies have demonstrated that age-inappropriate expansion of CD4^+^CD28^−^ T cells also occurs in patients with autoimmune disorders and in patients at risk for inflammatory vascular complications. This suggests that these cells might contribute to disease pathogenesis^63^. A major cause of CD28 loss in T cells is replicative senescence caused by repeated antigenic stimulation, although CD28 expression is also gradually lost when cells are exposed to the proinflammatory cytokine milieu^37, 38, 63^. Of interest, chronic exposure to TNF-α leads to downregulation of the CD28-specific initiator complex, resulting in decreased CD28 expression on CD4^+^ T cells^64, 65^. Therefore, TNF-α blocking agents have been considered as possible therapeutics to diminish pathogenic CD4^+^CD28^−^ T cells in rheumatoid arthritis^63^. Since repeated antigenic stimulation leads to chronic inflammation, making proinflammatory cytokines such as TNF-α abundant, loss of CD28 due to both replicative senescence and cytokine exposure might not be mutually exclusive in inflammatory disorders. In this context, it should be noted that IS markedly increased monocyte TNF-α and IL-1β production as shown in our *in vitro* culture data (Fig. 1D and Suppl. Fig. 2A and B), and CD4^+^CD28^−^ T cells in ESRD have immunophenotypes typical of senescent T cells, such as gain of CD57 and CD85j and loss of IL-7Rα, indicating that they have undergone extensive cell division (Suppl. Fig. 8C). More importantly, chronic exposure to TNF-α allows for gradual loss of CD28 expression on TCR-stimulated, purified CD4^+^CD28^+^ T cells from ESRD patients (Fig. 5B and C). Thus, this could be one mechanism for expansion of CD4^+^CD28^−^ T cells in patients with ESRD.

Senescent CD4^+^CD28^−^ T cells possess unique features characteristic of cytotoxic cells (like CD8^+^ T cells) (Fig. 6C and D)^11, 66^, recruitment of these cells by CX3CL1 likely results in damage to the vascular endothelium via induction of apoptosis (Fig. 6A and B). Indeed, CD4^+^CD28^−^ T cells were reported to be a major T cell subset observed among infiltrating cells at sites of inflammation and were found to be associated with atherosclerosis, suggesting that CD4^+^CD28^−^ T cells take part in vascular plaque destabilization^22^.

Taken together, the findings presented in this study demonstrate that IS, a major uremic toxin, induces TNF-α production in monocytes through AhR pathway and an increased TNF-α is involved with the expansion of CD4^+^CD28^−^ T cells and their recruitment to activated vascular endothelial cells. These changes critically affect the pathogenesis of cardiovascular diseases in ESRD. Thus, this suggests that IS-mediated immune dysfunction is an important contributor to the development and progression of CVD in patients with ESRD.

## Methods

### Human subjects and cell isolation

The study protocols were approved by the institutional review board of Seoul National University Hospital and Severance Hospital. Peripheral blood of ESRD patients and healthy controls (HCs) was drawn after obtaining written, informed consent. The methods were performed in accordance with the approved guidelines. Peripheral blood mononuclear cells (PBMC) were isolated from blood by density gradient centrifugation (Bicoll separating solution; BIOCHROM, Cambridge, UK). Total monocytes were negatively separated from PBMC with pan-monocyte microbeads (Miltenyi Biotec, Auburn, CA), if no special mention in the figure legend. Total CD4^+^ T cells and CD4^+^ memory T cells were negatively enriched from PBMC using human CD4 T cell enrichment kit and human memory CD4 T cell enrichment kit (STEMCELL Technologies, Vancouver, Canada), respectively. CD4^+^CD28^+^ and CD4^+^CD28^−^ T cells were separated from CD4^+^ memory T cells using a human CD28 microbead kit (Miltenyi Biotec) as described in user’s instructions. CD28^−^ cells were retained in the run-through fraction including CD28 unlabeled cells.

### Flow cytometric analysis

The following antibodies were used for flow cytometric analysis: Anti-Perforin-Alexa fluor 488, anti-CD28-allophycocyanin (APC), anti-CD19-APC, anti-CD3-APC, anti-CD56-APC, anti-CD3-APC-cyanin7 (Cy7), anti-CD8-APC-Cy7, anti-CD14-APC-Cy7, anti-CD14-fluorescein isothiocyanate (FITC), anti-HLA-DR-FITC, anti-CD16-R-phycoerythrin (PE), anti-GATA3-PE, anti-CD45RA-PE-cyanin 5 (Cy5), anti-CD4-PE-Cy5, anti-CD16-PE-Cy5, anti-CCR7-PE-Cy7, anti-CD4-PE-Cy7, anti-CD4-V450, anti-GranzymeB-V450, anti-CD8-V500, anti-CD3-V500 (all from BD Bioscience, Franklin Lakes, NJ), anti-CD57-FITC, anti-CD4-FITC, anti-CD85j-PE, anti-CX3CR1-PE, anti-T-bet-PE-Cy7, anti-Eomes-Peridinin chlorophyll (PerCP)-efluor710 (six from eBioscience, San Diego, CA), anti-HLA-DR-PE-Cy5, anti-IL-7Rα-V450, anti-CD57-V450 (three from BioLegend, San Diego, CA), anti-CX3CR1-FITC (MBL International Corporation, Woburn, MA). For intracellular staining of T cell lineage-specific transcription factors (T-bet, Gata3, and Eomes), granzyme B and perforin, PBMC were fixed and permeabilized with Fix/Perm buffer set (BioLegend). Stained cells were acquired by a BD LSRFortessa (BD bioscience) and analyzed by using FlowJo software (ver. 9.0 or 10.0; Tree Star, OR).

### Measurement of indoxyl sulfate and *p*-cresyl sulfate in human plasma

Simultaneous quantification of indoxyl sulfate and *p*-cresyl sulfate in human plasma were analyzed using liquid chromatography–tandem mass spectrometry (LC–MS/MS)^67^. In brief, plasma samples (25 μl) were prepared by protein precipitation with 225 μl of internal standard solution (indoxyl sulfate_d4, 100 ng/ml in 100% acetonitrile) followed by centrifugation at 19,500 × g for 10 min at 4 °C. The supernatant (20 μl) was mixed with 980 μl of distilled water and 3 μl was injected into a Kinetex 2.6 μm C18 column (2.1 × 100 mm, Phenomenex, USA). The mobile phase consisted of solvent (A) 5 mmol/L ammonium acetate solution and solvent (B) 100% methanol. The flow rate was 0.2 ml/min with a total cycle time of 12 min/sample. The initial gradient condition was 20% B (8/2, v/v) for 1 min followed by a linear gradient up to 60% B over the next 1.5 min, followed by 95% B over the next 0.5 min. It was returned to 20% B over the next 0.5 min, followed by 20% B for 9 min. Indoxyl sulfate and *p*-cresyl sulfate were eluted at 1.97 min and 3.45 min, respectively. The calibration standards for indoxyl sulfate and *p*-cresyl sulfate were linear over the range of 0.2 to 50 μg/ml and 0.4 to 80 μg/ml, respectively. The human blank plasma was treated with active charcoal to eliminate endogenous metabolites. Selected reaction monitoring analysis was performed using a triple quadrupole mass spectrometer (API4000, AB SCIEX, Foster City, CA, USA) equipped with an ESI source. Indoxyl sulfate and *p*-cresyl sulfate were detected at m/z 211.921 → 79.700, 186.947 → 106.900, respectively, in multiple reactions monitoring positive mode.

### Cell culture

Purified monocytes and CD4^+^ T cells derived from HCs were cultured in RPMI 1640 medium supplemented with 10% fetal bovine serum (FBS), 100 units/ml penicillin, 100 μg/ml streptomycin, and 2 mM L-glutamine. Monocytes were seeded at 5 × 10^5^ into polypropylene round bottom tubes (BD Bioscience) in the presence of various concentrations of IS (Sigma-Aldrich, St. Louis, MO). In some experiments, monocyte-conditioned media (MCM) was prepared after incubation for 48 hr with IS. For experiments using pooled uremic serum, purified monocytes were incubated for 24 hr with pooled serum from ESRD patients or age-matched HCs. The cells and supernatant were harvested for further analysis.

HUVECs, human umbilical vein endothelial cells, were purchased from Lonza (Basel, Switzerland) and were grown adherent to 0.1% gelatin-coated cell culture dishes (Welgene, Gyeongsan-shi, Republic of Korea) with EGM^TM^-2 basal medium (Lonza) supplemented with 2% fetal bovine serum (FBS), 0.4% hFGF-β, 0.1% hEGF, VEGF, R3-IGF-1, ascorbic acid, heparin, gentamicin/amphotericin-B, and 0.04% hydrocortisone. HUVECs were seeded at 0.1% on a gelatin-coated six well, flat-bottom plate (BD Bioscience) and stimulated with recombinant human TNF-α (1 to 10 ng/ml; R&D systems) or recombinant human IL-1β (10 ng/ml; R&D systems) for the indicated time periods.

For long term *in vitro* culture of CD4 T cells, purified CD4^+^CD28^+^ T cells were stimulated with anti-CD3/CD28 Ab coated beads (Dynabeads® T-Activator CD3/CD28; Thermo Fisher Scientific, Waltham, MA) and IL-2 (50 U/ml; PeproTech, Rocky Hill, NJ) in the absence or presence of TNF-α (10 ng/ml; R&D systems, Minneapolis, MN). At 4 days, beads were removed using a magnet and the cytokines were re-supplemented every 3–4 days. CD28 expression was assessed every 7 days using a BD LSRFortessa.

### Transfection of AhR-targeted siRNA

Purified primary total monocytes were transfected with siRNA of AhR or negative control (NC) (Dhamacon, SMART pool ON-TARGET plus human AhR siRNA or non-targeting siRNA) using lipofectamine imax (Invitrogen). After 24 hr, the cells were treated with IS for another 24 hr.

### Enzyme-linked immunosorbent assay (ELISA)

The amounts of TNF-α and IL-1β in culture supernatants of IS-treated monocytes were quantified using commercial human ELISA kits (Both from eBioscience). The amount of CX3CL1 produced by TNF-α-treated HUVECs was quantified in the culture media using human CX3CL1/Fractalkine ELISA kit (R&D systems). The measurement of OD (Optical density value) was performed by Infinite M200 (Tecan, Männedorf, Switzerland).

### Quantitative RT-PCR

cDNA was synthesized from total RNA, and real-time quantitative RT-PCR was performed in triplicate on a 7500 PCR system (Applied Biosystems by Life Technologies Corp, Waltham, MA) using the SensiFAST SYBR® No-ROX (Bio-line, London, UK) as previously described^21^. The sequences of the sense and antisense primers used in this study were as follow; TNF-α: 5′-AGCCCATGTTGTAGCAAACC-3′ and 5′-TGAGGTACAGGCCCTCTGAT-3′; IL-1β: 5′-CACGATGCACCTGTACGATCA-3′ and 5′-GTTGCTCCATATCCTGTCCCT-3′; CX3CL1: 5′-TCCTTACCAGCAGAGCACCT-3′, 5′-GTCTCTGCTCTGCCCATTTC-3′; AhR: 5′-CCG TGT CGA TGT ATC AGT GC-3′ and 5′-GCC TGG CAG TAC TGG ATT GT-3′; CYP1A1: 5′-TCTTCCTTCGTCCCCTTCAC-3′ and 5′-TGGTTGATCTGCCACTGGTT-3′ and CYP1B1: 5′-TGCCTGTCACTATTCCTCATGCCA-3′ and 5′-ATCAAAGTTCTCCGGGTTAGGCCA-3′; and β-actin: 5′-GGACTTCGAGCAAGAGATGG-3′ and 5′-AGCACTGTGTTGGCGTACAG-3′. The levels of gene expression were normalized to the expression of *ACTINB*. The comparative C_T_ method (*ΔΔ*C_T_) was used for the quantification of gene expression.

### Western blot analysis

Cell lysates were prepared from freshly-isolated monocytes. To analyze the expression of AhR in these cells, whole cell lysates were separated on an 8% SDS-polyacrylamide gel and blotted onto a polyvinylidene difluoride (PVDF) membrane (Bio-Rad, Hercules, CA), The membrane was incubated overnight at 4 °C with rabbit anti-human AhR polyclonal Ab (Cell Signaling Technology), followed by incubation with the HRP-conjugated secondary Ab for 1 hour. The membranes were developed by SuperSignal West Femto Maximum Sensitivity substrate kit (Thermo scientific, Waltham, MA).

### Chemotaxis assay

Purified CD4^+^ memory T cells derived from ESRD patients were stained with anti-CD28-APC (BD Biosciences) for 30 min at 4 °C. Chemotaxis of stained CD4^+^ memory T cells was analyzed using 24-well Transwell chambers with 5 μM pores (Corning Inc, Corning, NY). 3 × 10^5^ cells in 100 μl chemotaxis buffer (RPMI 1640 with 0.5% BSA) were placed in the upper chambers. Recombinant human CX3CL1 (2.5 to 20 ng/ml; R&D systems) in 600 μl chemotaxis buffer was placed in the lower wells and the chambers were incubated for 2 hours at 37 °C and 5% CO_2_. Migrated cells located in the bottom wells were collected and analyzed using a BD LSRFortessa.

### TUNEL assay

HUVECs were stimulated with recombinant human IFN-γ (2,000 U/ml; eBioscience) for 48 hours to induce MHC Class II expression. The medium was removed and EBM-2 complete medium added in the presence of superantigens, SEB (10 ng/ml) and TSST-1(10 ng/ml; both from Toxin technology, Sarasota, FL). After 1 hour of loading with superantigens, CD4^+^CD28^−^ or CD4^+^CD28^+^ T cells were added to the HUVECs and were co-cultured for 4 hours. Apoptotic HUVECs following co-culture with CD4^+^CD28^−^ T cells were detected using the *In Situ* Cell Death Detection Kit, TMR red (Roche, Indianapolis, IN) as described in the instructions. The red apoptotic cells were visualized on a fluorescence microscope using the Leica DMI6000B (Leica camera, Wetzlar, Germany).

### Statistical analysis

A two-tailed paired or unpaired student’s t-test was done to analyze data using Graph Pad Prism 5 (GraphPad Software, La Jolla, CA) and Microsoft Excel 2013. *P* values of less than 0.05 were considered statistically significant.

## Electronic supplementary material

Supplementary information
