# Supplementary material for: Indoxyl sulfate (IS)-mediated immune dysfunction provokes endothelial damage in patients with end-stage renal disease (ESRD)
Source: Sci Rep. 2017 Jun 8;7:3057. doi: 10.1038/s41598-017-03130-z (PMC5465082; doi:10.1038/s41598-017-03130-z)
Supplement: Supplementary file 1 — Supplementary information [file 41598_2017_3130_MOESM1_ESM.pdf]

Title: Indoxyl sulfate (IS)-mediated immune dysfunction provokes endothelial damage in patients with end-stage renal disease (ESRD)

Authors: Hee Young Kim, Tae-Hyun Yoo, Yuri Hwang, Ga Hye Lee, Bonah Kim, Ji yeon Jang, Hee Tae Yu, Min Chang Kim, Joo-Youn Cho, Chan Joo Lee, Hyeon Chang Kim, Sungha Park and Won-Woo Lee

Supplementary Material and Methods

Measurement of ROS

Cells were incubated with 10 μM 5-(and-6)-chloromethyl-2′,7′-dichlorodihydrofluorescein diacetate, acetyl ester (CM-H_2DCFDA; Invitrogen, Carlsbad, CA) for 30 min, followed by washing with DPBS, and were treated for the indicated times with IS in the presence of NAC (N-acetyl-cysteine; Sigma-Aldrich, St. Louis, MO), ROS inhibitor or GNF351 (Calbiochem, San Diego, CA), AhR antagonist. ROS production by the cells was analyzed using flow cytometry.

Supplementary figures

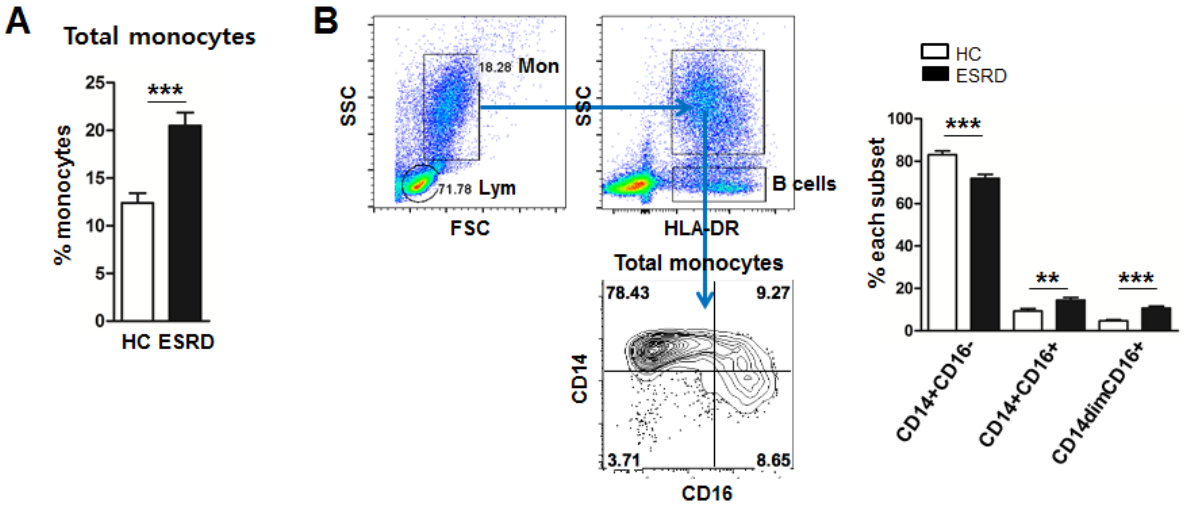

Supplementary figure 1. CD16$^+$ monocytes are significantly expanded in ESRD patient.

(A) Frequencies (%) of total monocytes (defined as HLA-DR$^+$CD14$^{dim/+}$) among PBMCs were analyzed in ESRD patients (n=50) and age-matched HCs (n=28). Monocytes were defined as cells expressing HLA-DR, but not lineage markers, such as CD3 (T cells), CD19 (B cells), or CD56 (NK cells) by flow cytometry. **(B)** Frequencies (%) of three distinct monocyte subsets were compared between ESRD patients and HCs. Total monocytes were further subdivided into three subsets by their expression of CD14 and CD16. Representative contour plot of peripheral monocytes from an ESRD patient. Bar graphs show the mean ± SEM. ** = p<0.01, and *** = p<0.001 by two-tailed unpaired t-test.

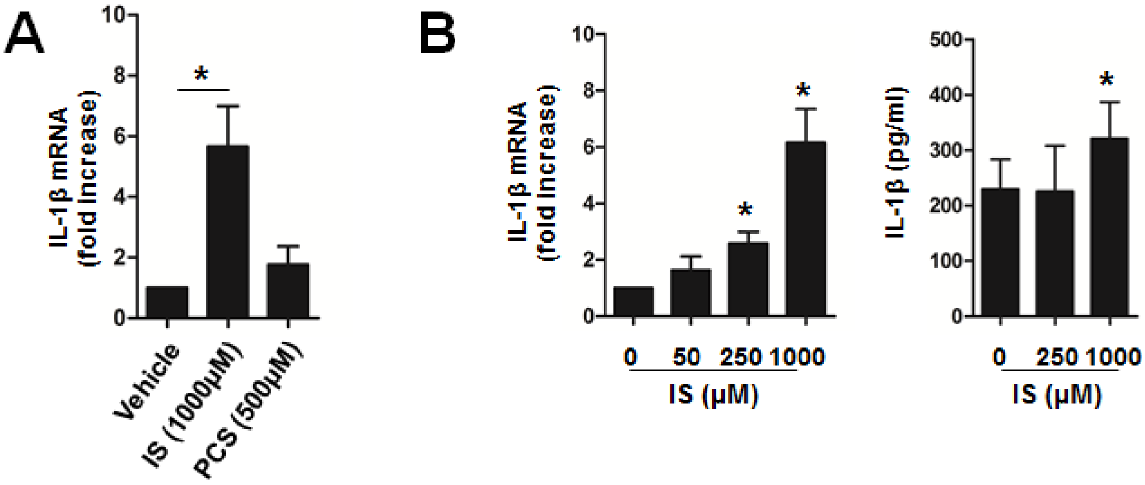

Supplementary figure 2. Indoxyl sulfate (IS) is responsible for enhanced IL-1β expression by human monocytes.

(**A**) Purified monocytes were stimulated with 1,000 μM of IS or 500 μM of PCS for 24 hr and then IL-1β mRNA expression was analyzed by real-time RT-PCR. (**B**) Purified monocytes were stimulated with IS at the indicated concentrations. The expression of IL-1β mRNA was analyzed by real-time RT-PCR after a 24 hr stimulation (**left**) and its protein level was quantified by ELISA at 48 hr post-stimulation (**right**). Expression of β-actin was used as a normalization control for real-time RT-PCR analysis. Bar graphs show the mean ± SEM of three (**A**) and five to seven (**B**). * = $p<0.05$ by two-tailed paired *t*-test (**A** and **B**).

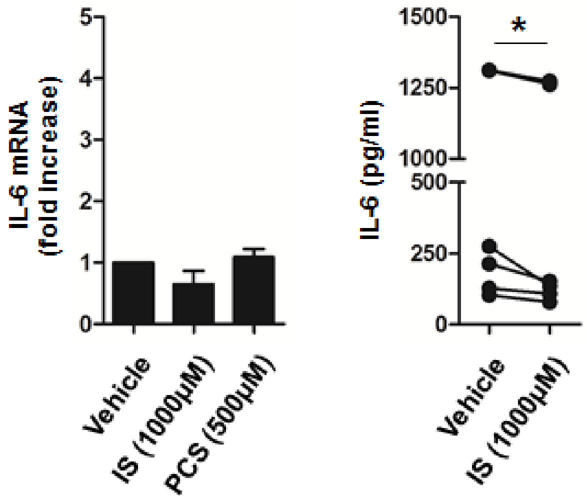

Supplementary figure 3. No induction of IL-6 is observed in monocytes treated with both IS and PCS.

(**Left**) Purified monocytes were stimulated with 1,000 μM of IS or 500 μM of PCS for 24 hr and then IL-6 mRNA expression was analyzed by real-time RT-PCR. (**Right**) Purified monocytes were stimulated with 1,000 μM of IS. The amount of IL-6 in the culture supernatant was quantified by ELISA at 48 hr post-stimulation. Expression of β-actin was used as a normalization control for real-time RT-PCR analysis. Bar graphs show the mean ± SEM of three (**Left**) and six (**Right**). * = p<0.05 by two-tailed paired *t*-test.

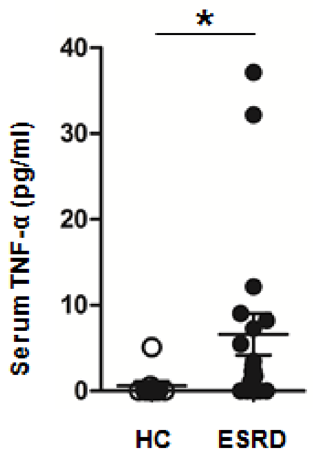

Supplementary figure 4. Serum TNF-a level is higher in ESRD patients than in healthy controls.

The amount of TNF-α in serum was quantified by ELISA and compared between ESRD patients (n=19) and age-matched HCs (n=10). * = p<0.05 by two-tailed unpaired t-test.

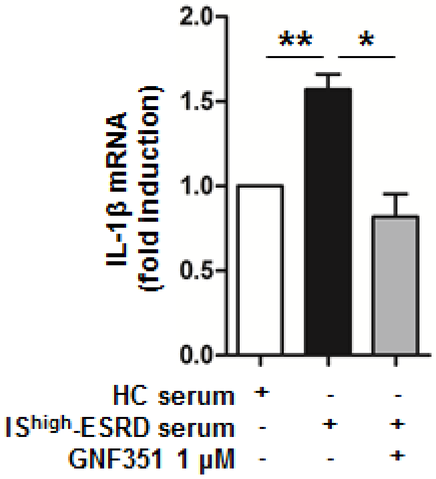

Supplementary figure 5. Uremic sera-activated induction of IL-1β is abrogated in monocytes by GNF351, AhR antagonists.

Sera were pooled from patients with the top 10 (IS^{higher}-ESRD) IS serum concentrations. As a control, sera from healthy controls were pooled. Monocytes isolated from healthy controls were treated with 30% (v/v) of the indicated sera for 24 hr with or without 1 μM of GNF351, AhR antagonists. Gene expression levels were analyzed by real-time RT-PCR. Expression of β-actin was used as a normalization control. Bar graphs show the mean ± SEM of four to six independent experiments. * = p<0.05 and ** = p<0.01 by two-tailed paired t-test.

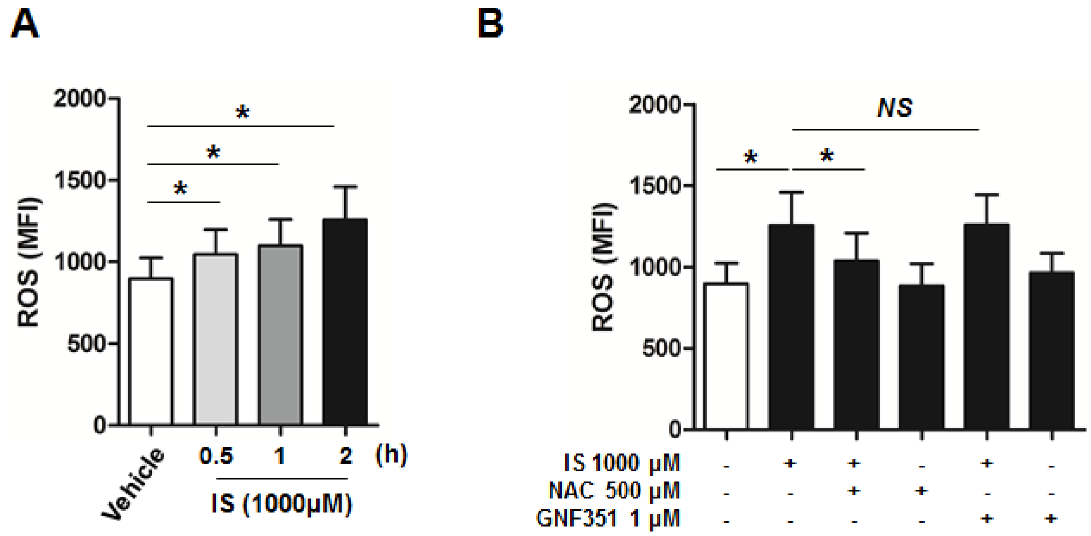

Supplementary figure 6. Purified monocytes were incubated with 10 µM 5-(and-6)-chloromethyl-2′,7′-dichlorodihydrofluorescein diacetate, acetyl ester (CM-H_2DCFDA, Invitrogen) for 30 min and were washed with DPBS. The monocytes were treated for 2 hr with IS in the presence of NAC (N-acetyl-cysteine), ROS inhibitor or GNF351, AhR antagonists. The level of ROS production was analyzed using flow cytometry. Bar graphs show the mean ± SEM of four independent experiments (**A** and **B**). * = p<0.05 by two-tailed paired t-test. *NS* indicates not significant.

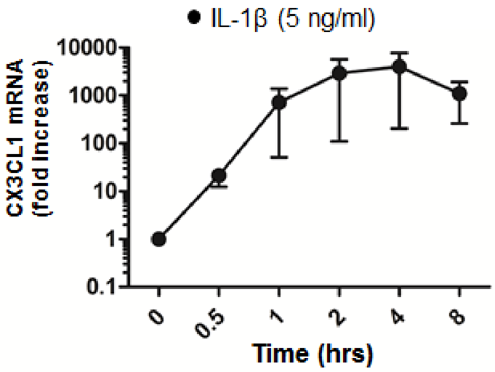

Supplementary figure 7. IL-1β markedly upregulates CX3CL1 production by HUVECs

HUVECs were stimulated with IL-1β (5 ng/ml) for up to 8 hours and CX3CL1 mRNA expression was analyzed by real-time RT-PCR at the indicated time-points. Expression of β-actin was used as a normalization control. Bar graphs show the mean ± SEM of three independent experiments.

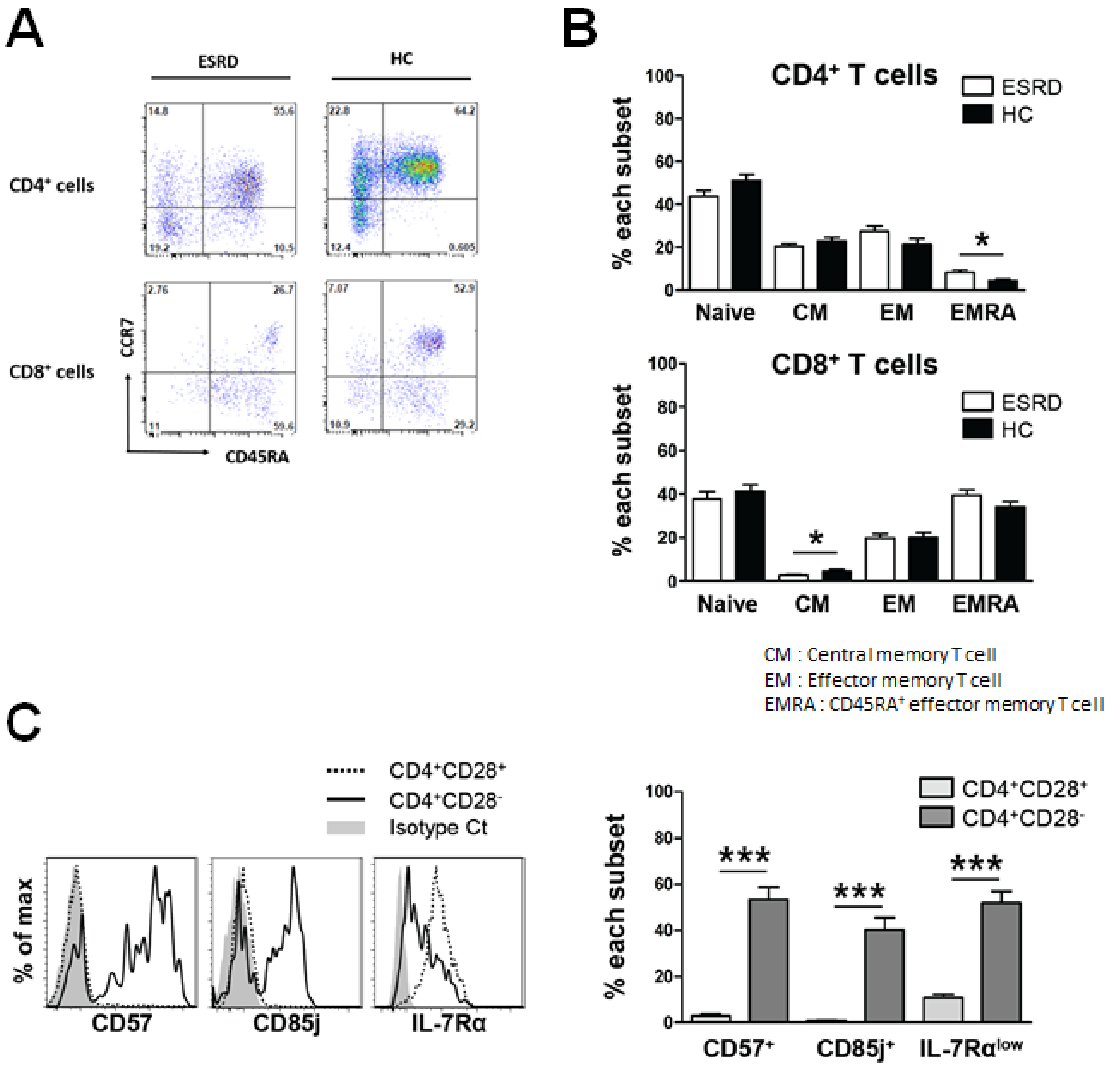

Supplementary figure 8. Phenotypic characterization of T cells in patients with ESRD

Phenotypic characterization of peripheral T cells in peripheral blood mononuclear cells (PBMCs) of ESRD patients (n=50) and age-matched HCs (n=28) by flow cytometry. **(A)** Distribution of functional T-cell subsets defined by the expression pattern of CD45RA and CCR7 in $CD4^+$ and $CD8^+$ T cells. Naive ($CD45RA^+CCR7^+$), central memory (CM: $CD45RA^+CCR7^+$), effector memory (EM: $CD45RA^-CCR7^-$), and $CD45RA^+$ effector memory (EMRA: $CD45RA^+CCR7^-$). **(B)** Frequencies (%) of $CD28^-$ cells in $CD4^+$ and $CD8^+$ T cells. **(C)** Representative flow cytometric analysis of senescence marker (CD57, CD85j, and IL-7Rα) expression on $CD4^+CD28^+$ and $CD4^+CD28^-$ T cells. **(D)** Frequency (%) of $CD57^+$, $CD85j^+$, and IL-7Rα^{low} cells in $CD4^+CD28^+$ and $CD4^+CD28^-$ T cell populations (n=24 or 32) of ESRD patients. Bars graphs show the mean ± SEM. * = p<0.05, ** = p<0.01, and *** = p<0.001 by two-tailed unpaired (**A** and **B**) or paired t-test (**C**).
